# Intradural Cauda Equina Metastasis of Colorectal Adenocarcinoma: A Case Report

**DOI:** 10.7759/cureus.81130

**Published:** 2025-03-25

**Authors:** Chung-Hua Chu, Wen-Tien Wu, Kuang-Ting Yeh

**Affiliations:** 1 Department of Orthopedic Surgery, Taipei Tzu Chi Hospital, New Taipei City, TWN; 2 Department of Orthopedic Surgery, Hualien Tzu Chi Hospital, Hualien, TWN

**Keywords:** cauda equina metastasis, colorectal carcinoma, durotomy, intradural spinal metastasis, spine oncology

## Abstract

Intradural spinal metastasis of non-neurogenic tumors is rare. Even more uncommon is intradural metastasis involving the cauda equina. Among these, colorectal adenocarcinoma metastasizing to the cauda equina is exceedingly rare, with no previously documented cases.

We report a case of a 44-year-old male with a history of descending colon adenocarcinoma, initially treated with laparoscopic resection in 2011, followed by radical proctectomy for recurrent sigmoid adenocarcinoma in 2013. Over time, he developed progressive left-sided sciatica and foot drop. Imaging revealed an intradural extramedullary lesion at the cauda equina. The patient underwent L4-S2 left hemilaminectomy and S2 foraminectomy, with histopathology confirming metastatic adenocarcinoma. His postoperative recovery was uneventful, with significant pain relief and partial motor function improvement. However, local recurrence occurred at 16 months postoperatively, and the patient passed away 5.8 years after surgery.

This case represents the first reported instance of intradural cauda equina metastasis from colorectal adenocarcinoma. The presumed metastatic route was via the Batson venous plexus, as systemic metastases were absent. Despite surgical resection improving symptoms, the long-term prognosis remains poor. Contrast-enhanced T1-weighted MRI is crucial for diagnosis, and total resection may contribute to prolonged survival in select patients.

## Introduction

Intradural spinal metastasis of non-neurogenic tumors is rare, representing 4-6% of all spinal metastasis [1]. These metastases can occur at any level of the spine, with approximately 130 cases reported in the literature [2]. In particular, only five cases of intradural metastasis originating from the colorectal have been documented [3-6].

Tumors occurring in the cauda equina are predominantly primary and benign, including neurofibroma, ependymoma, hemangioblastoma, astrocytoma, lipoma, and epidermoid tumor [7]. In contrast, cauda equina metastasis is even rarer [8]. To date, only 55 cases of non-neural intradural extramedullary metastasis involving the cauda equina have been reported [9]. The most commonly affected primary tumor sites are the lung, kidney (renal), and breast [9].

Colorectal carcinoma (CRC) is a highly metastatic disease. The most common metastatic sites are regional lymph nodes, liver, lung, and peritoneum [10]. Besides bone, many other places, including the brain, are also involved. Brain metastasis of CRC is not common [10], intramedullary spinal cord lesions are rare, and intradural extramedullary cauda equina metastasis, to our best knowledge, is the first reported case.

## Case presentation

**Figure 1:**
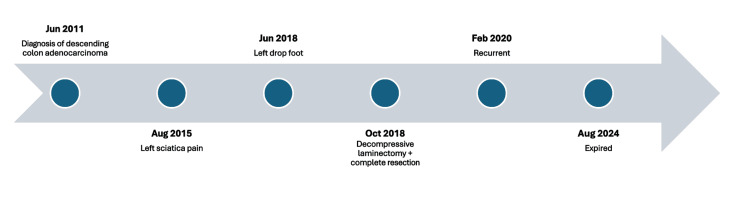
Timeline of disease progression and treatment.

This 44-year-old patient was referred to the orthopedic department in June 2018 due to progressive weakness in the left foot dorsiflexion (Figure 1). He had experienced numbness and pain in the left inguinal region and scrotum for three years, along with left buttock, posterior thigh, and calf, extending to the lateral foot that began in August 2015.

His medical history included a diagnosis of descending colon adenocarcinoma (cT3N0M0) in June 2011, for which he underwent laparoscopic anterior resection five days after diagnosis.

In October 2012, he developed changes in bowel habits and hematochezia, leading him to visit our outpatient department in March 2013. He was diagnosed with recurrent sigmoid adenocarcinoma and subsequently underwent radical proctectomy with low anterior resection, followed by six months of postoperative concurrent chemoradiotherapy (CCRT).

**Figure 2:**
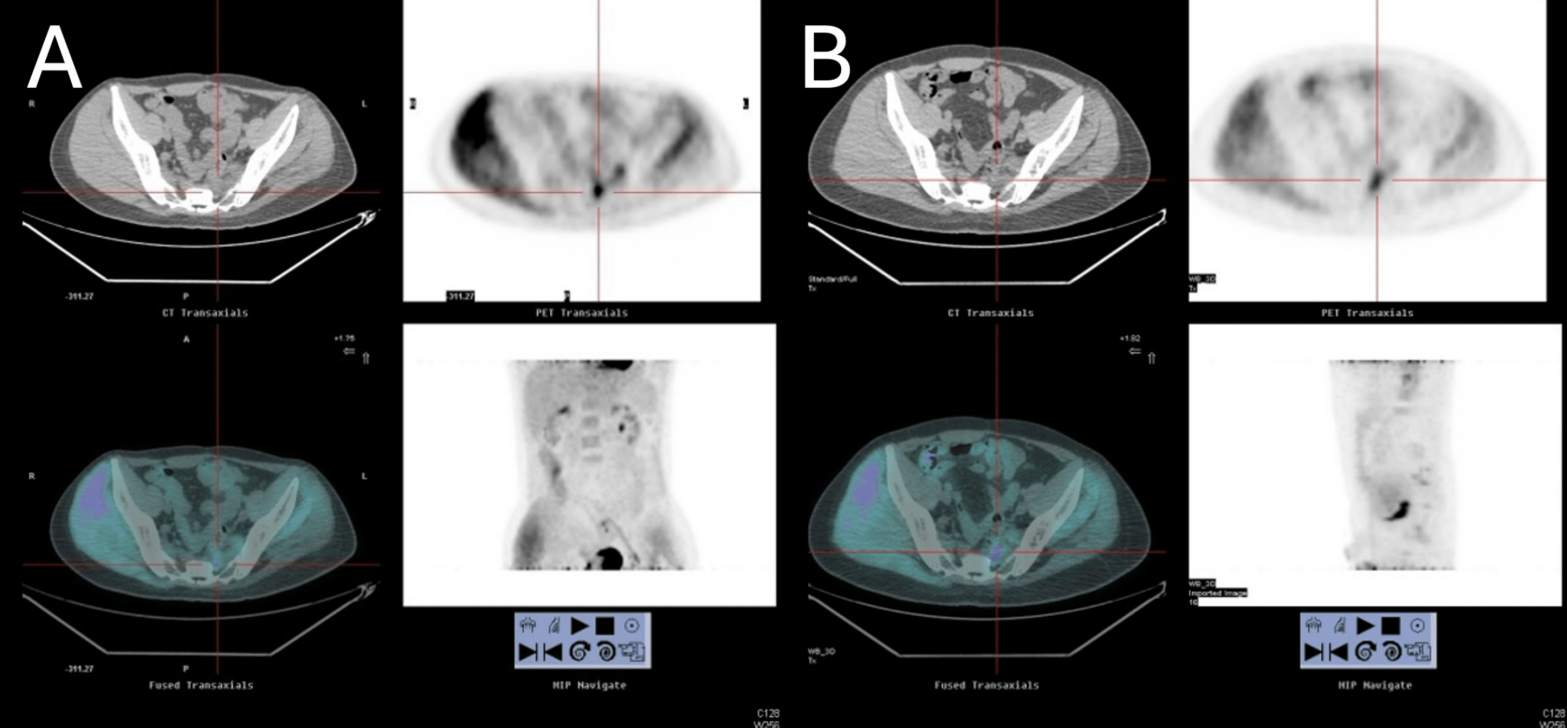
(A) A hypermetabolic lesion in the presacral region with increased fluorodeoxyglucose (FDG) uptake. (B) Another imaging reconstruction or different slice positioning, reinforcing the metabolic activity and anatomical location of the lesion.

**Figure 3:**
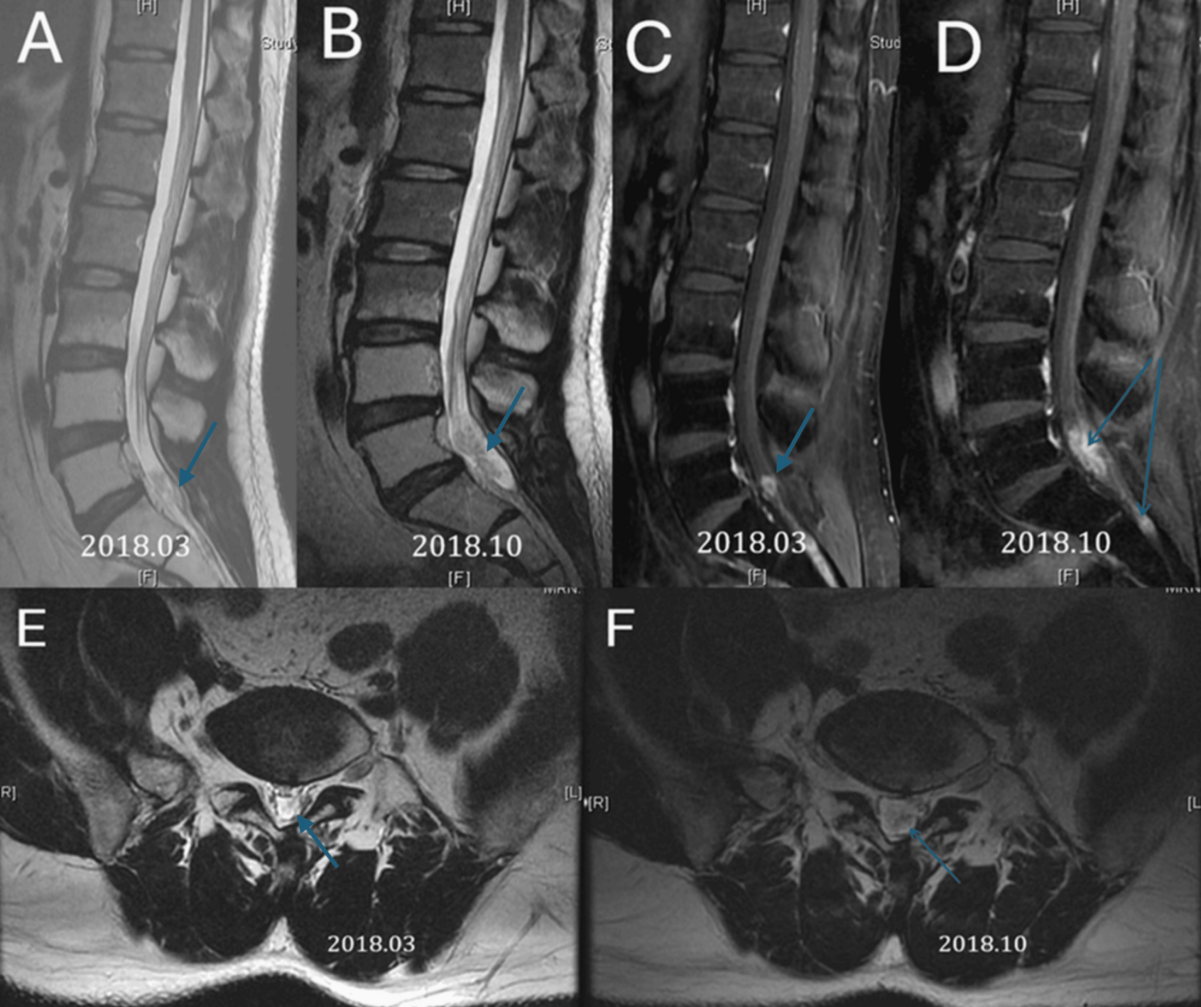
(A) T2-weighted MRI: An intradural hyperintense lesion is observed in the cauda equina region, causing mild compression of the nerve roots. At this stage, the patient experienced left-sided sciatica. (B) T2-weighted MRI reveals further enlargement of the lesion, with increased compression of the cauda equina nerve roots and more pronounced spinal canal narrowing. The patient's symptoms worsened, with progressive left foot drop and increased pain, prompting the decision for surgical intervention. (C-D) Contrast-enhanced T1-weighted MRI. (E-F) Axial T2-weighted MRI showing an intradural lesion compressing the cauda equina nerve roots.

Between 2016 and 2018, the patient's left-sided sciatica worsened, with increased severity and frequency of pain. PET and MRI scans (Figures 2, 3) were performed. The imaging revealed a heterogeneous enhancing lesion in the presacral space, invading the left S2 nerve root. Suspecting tumor recurrence, he was started on oral chemotherapy with UFUR (tegafur/uracil) for three months, followed by continued treatment with Xeloda. In the two months leading up to his admission, his symptoms progressively worsened, with increasing left foot pain and a developing left foot drop.

Neurological examination revealed significant weakness in the left ankle dorsiflexion and plantar flexion (0/5), left leg atrophy, saddle anesthesia, and loss of the Achilles reflex. However, there was no bladder or bowel dysfunction.

After receiving a diagnosis of cauda equina syndrome, the patient was informed that surgery would likely serve a palliative purpose, aimed at improving quality of life by relieving pain, preserving walking ability, and preventing further neurological decline. Although bladder and bowel function were intact, the patient expressed a strong desire to maintain independence in daily activities and minimize future disability. After discussing treatment options, risks, and benefits with the surgical team, the patient opted for surgical intervention. Under this shared decision-making process, the patient underwent L4-S2 left hemilaminectomy and left S2 foraminectomy in October 2018.

**Figure 4:**
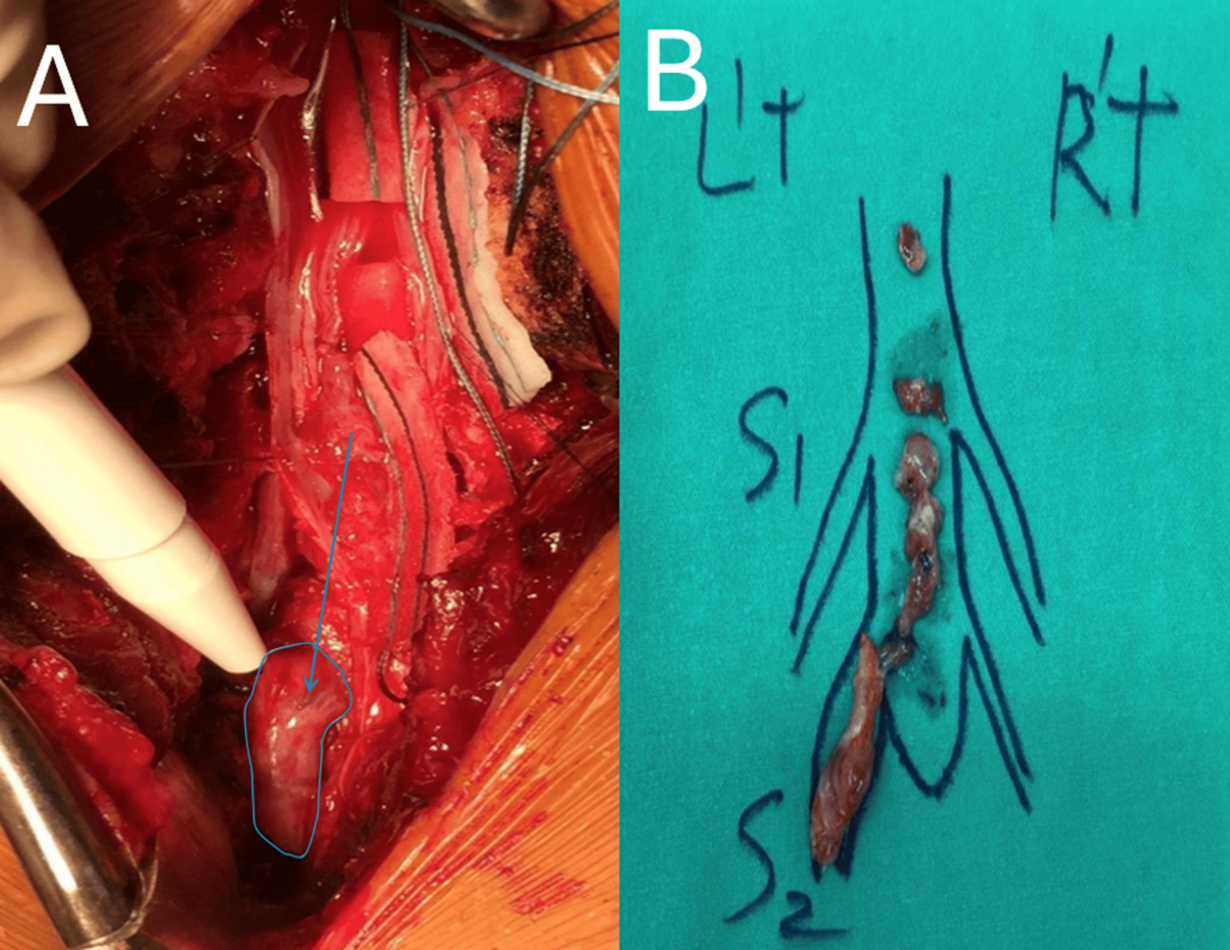
(A) Intraoperative photograph showing the exposed cauda equina after durotomy. The intradural tumor (marked by the arrow) is visible, compressing the nerve roots. (B) Resected tumor specimens laid out on a surgical drape, arranged according to their anatomical location, with an illustration indicating the S1 and S2 nerve roots for reference.

Intraoperatively, a reddish-white hemorrhagic lesion was observed in the caudal rootlets, with some rootlets directly infiltrating the tumor (Figure 4). Careful dissection revealed a well-encapsulated, denatured, hypertrophic, and pathologic mass involving the left S2 nerve root. Despite efforts to preserve the nerve root, the left S2 root was ultimately sacrificed, and the tumor was completely excised. No evidence of bony metastasis was found. Pathologic examination confirmed metastatic adenocarcinoma.

The patient's postoperative course was uneventful. During the one-, three-, and six-month OPD follow-ups, the patient experienced complete resolution of left foot pain, improvement in muscle power of left dorsiflexion and plantar flexion to 3/5, and was able to ambulate without assistance. Imaging studies, including brain MRI, whole-body PET scan, chest CT, and abdomen-to-pelvis CT, showed no other metastasis.

**Figure 5:**
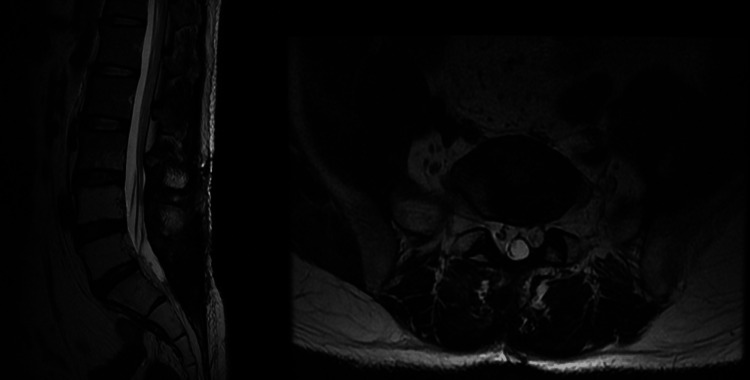
No recurrence at 10 months postoperatively.

**Figure 6:**
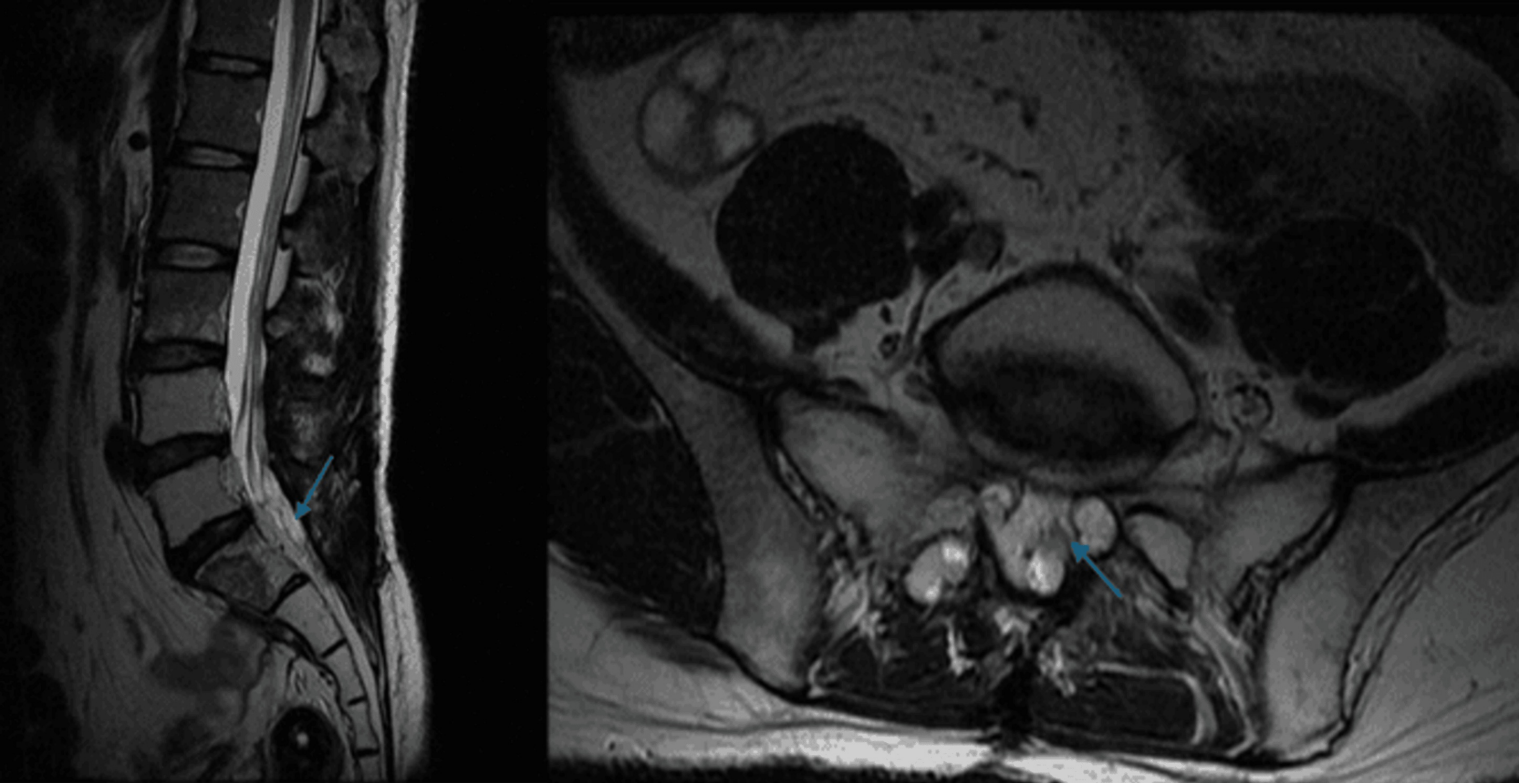
Recurrence was detected at 16 months postoperatively.

In August 2019, MRI at 10 months postoperatively showed no recurrence (Figure 5). However, by February 2020, recurrence was detected (Figure 6). The patient opted for continued conservative treatment but unfortunately expired in August 2024.

## Discussion

Colorectal cancer is a highly metastatic disease. It accounts for 10-15% of distal metastasis. The most common sites are the liver (20-30%) and lungs (10-20%) [11]. Brain metastasis is rare, developing in about 1-4% of cases [12], but is concomitant with lung metastasis in 55% to 85% of patients and liver metastases in up to 75%.

Intradural extramedullary metastasis is rare and has only 130 cases reported [2]. The most common primary sites of metastasis are the lung (26.2%), renal (26 20%), breast (13.8%), and prostate (8.5%) [2]. Only five cases of colorectal cancer have been reported so far [3-6]. To our best knowledge, our case is the first report of intradural cauda equina metastasis from colorectal cancer.

To date, five routes for the metastasis of intradural spinal tumors from non-neurogenic origins have been reported: (a) drop metastasis via the subarachnoid space; (b) hematogenous spread via the arterial system; (c) venous dissemination through the Batson plexus, a rich venous network; (d) perineural lymphatic spread; and (e) direct invasion from involved osseous structures into the cerebrospinal fluid through the dura [2]. Perrin et al. [4] reported that 90% of intradural metastases occur in patients with intracranial metastases. However, this is unlikely in our case, as the brain MRI showed no evidence of metastasis. In colorectal cancer, the most common metastatic sites include the regional lymph nodes, liver, lung, and peritoneum [10]. In our case, lung CT showed no abnormalities, reducing the likelihood of hematogenous arterial spread. Additionally, PET imaging showed no evidence of lymph node absorption and no epidural metastasis, making lymphatic spread less probable. Direct invasion through osseous structures is also unlikely, as the whole-body bone scan revealed no metastatic lesions. The Batson plexus is an anastomosing venous network connecting the pelvis and spinal cord, with multiple collateral branches linking the epidural and intradural spaces. When intra-abdominal pressure increases, venous emboli can migrate to the spinal cord through valveless small veins, bypassing the lungs. Thus, in our case, the most likely mechanism of tumor spread is via the venous plexus.

Isolated intradural metastasis is like herniated intervertebral disc (HIVD), and is easily neglected [13]. However, when cramping pain occurs after light percussion or back pain is aggravated by position change, differential diagnoses more than HIVD should be suspected [14]. Our patient was diagnosed with HIVD initially. Besides, cauda equina lesions always occur many years after primary lesions are found. Symptoms of metastasis always progress faster than primary lesions [13,15]. From the onset of sciatica to the development of drop foot in cauda equina syndrome, our patient experienced a progression over 34 months.

Contrast-enhanced T1-weighted MRI is the primary diagnostic tool, allowing differentiation between single and multiple lesions, whether in the cauda equina or conus medullaris [16,17]. MRI can also detect leptomeningeal metastases and vertebral metastases [17]. Preoperatively, we utilized contrast-enhanced T1 MRI for diagnosis and surgical planning, while postoperative follow-up was conducted using non-contrast MRI.

Regarding the primary objectives of treating intradural cauda equina metastases, in addition to providing a pathological diagnosis, the main goals are to relieve the patient's pain, improve their quality of life, and restore their ambulation. However, because the tumor adheres tightly to the cauda equina nerve roots without a well-defined separation plane, complete resection may result in more severe complications [18]. Currently, the majority of published studies favor partial resection (accounting for over 80%), suggesting that complete resection may not be necessary [19]. Nevertheless, we endeavored to remove as much of the tumor as possible and sacrificed the S2 nerve root, yet no severe postoperative neurological deficits were observed.

Despite the availability of various treatment options, the prognosis of intradural cauda equina metastases remains poor and is largely dependent on the characteristics and behavior of the primary tumor [19]. Although most patients experience symptom improvement after surgery, a review by Palmisciano et al. involving 123 patients reported an average time to local recurrence of seven months and a mean overall survival of approximately 10 months. The median age of the patients in the cohort was 57 years [19]. This may be attributed to the fact that most cases occur in late-stage disease, with patients presenting with systemic metastases and a high tumor burden. In contrast, our patient experienced symptom improvement after surgery, local recurrence at 16 months, and survived 5.8 years after surgery. This prolonged survival may be related to the absence of systemic metastases, a relatively lower tumor burden, the fact that we performed a total resection, and the relatively young age of the patient (44 years old).

## Conclusions

Intradural extramedullary cauda equina metastasis from colorectal adenocarcinoma is definitely rare. This is the first reported case to date. Its management may be similar to that of other primary tumors, with laminectomy and resection potentially yielding favorable outcomes. However, local recurrence control time and overall survival remain limited. Therefore, when treating oncology patients, heightened vigilance is required when disproportionately severe symptoms appear. Earlier intervention and individualized treatment strategies should be developed and may lead to better prognostic outcomes.

## References

[1] Shin DA, Huh R, Chung SS, Rock J, Ryu S (2009). Stereotactic spine radiosurgery for intradural and intramedullary metastasis. Neurosurg Focus.

[2] Palmisciano P, Chen AL, Sharma M (2022). Intradural extramedullary spinal metastases from non-neurogenic primary tumors: a systematic review. Anticancer Res.

[3] Chow TS, McCutcheon IE (1996). The surgical treatment of metastatic spinal tumors within the intradural extramedullary compartment. J Neurosurg.

[4] Perrin RG, Livingston KE, Aarabi B (1982). Intradural extramedullary spinal metastasis. A report of 10 cases. J Neurosurg.

[5] Petterwood J, Lim K, Gonzalvo A, Quan G (2015). Intradural extramedullary colorectal adenocarcinoma metastasis to the cervical spine. ANZ J Surg.

[6] Wostrack M, Pape H, Kreutzer J, Ringel F, Meyer B, Stoffel M (2012). Surgical treatment of spinal intradural carcinoma metastases. Acta Neurochir (Wien).

[7] Ito K, Miyahara T, Goto T, Horiuchi T, Sakai K, Hongo K (2010). Solitary metastatic cauda equina tumor from breast cancer -case report. Neurol Med Chir (Tokyo).

[8] Mariniello G, Corvino S, Sgulò F, Guadagno E, Del Basso De Caro M, Maiuri F (2022). Intradural cauda equina metastases from renal cell carcinoma. Interdiscip Neurosurg.

[9] Pagano A, Iaquinandi A, Fraioli MF, Bossone G, Carra N, Salvati M (2023). Cauda equina syndrome from intradural metastasis of a non-neural tumor: case report and review of literature. Br J Neurosurg.

[10] Go PH, Klaassen Z, Meadows MC, Chamberlain RS (2011). Gastrointestinal cancer and brain metastasis: a rare and ominous sign. Cancer.

[11] Gofrit ON, Gofrit B, Goldberg SN, Popovtzer A, Sosna J, Hubert A (2024). The varied clonal trajectory of liver and lung metastases of colorectal cancer. Adv Cancer Biol Metastasis.

[12] Tan WS, Ho KS, Eu KW (2009). Brain metastases in colorectal cancers. World J Surg.

[13] Hassan O, Gassie K, Goyal A, Foskey S, Abode-Iyamah K (2023). Clinical features and neurosurgical management of metastatic intradural extramedullary renal cell carcinoma. Cureus.

[14] Hartvigsen J, Hancock MJ, Kongsted A (2018). What low back pain is and why we need to pay attention. Lancet.

[15] Kubota M, Saeki N, Yamaura A, Iuchi T, Ohga M, Osato K (2004). A rare case of metastatic renal cell carcinoma resembling a nerve sheath tumor of the cauda equina. J Clin Neurosci.

[16] Jawahar A, Ampil FL, Reddy PK, Hartman GH, Sathyanarayana S, Nanda A (2002). Analysis of outcome and prognostic factors in metastatic cauda equina compression: a 20-year single institution experience. Neurosurg Q.

[17] Löhr M, Tzouras G, Kocher M, Stenzel W, Reithmeier T, Klug N, Hampl JA (2009). Treatment strategies of space-occupying intradural metastases of the cauda equina of nonneurogenic origin. Acta Neurochir (Wien).

[18] Mackel CE, Alsideiri G, Papavassiliou E (2020). Intramedullary-extramedullary breast metastasis to the caudal neuraxis two decades after primary diagnosis: case report and review of the literature. World Neurosurg.

[19] Palmisciano P, Zaidi SE, Shlobin NA (2022). Intradural cauda equina metastases: a systematic review of clinico-radiological features, management, and treatment outcomes. Anticancer Res.

